# Determinants of the introduction of early complementary feeding before and after the third month of life: a multinomial analysis

**DOI:** 10.1590/1414-431X202010115

**Published:** 2020-11-18

**Authors:** T. Trovão, M.C.V. Cavalcante, M.C. Rodrigues, A.A. Ferraro, H. Bettiol, M.C.P. Saraiva, Z.C. Lamy, F. Lamy-Filho

**Affiliations:** 1 Programa de Pós-Graduação em Saúde Coletiva, Universidade Federal do Maranhão, São Luís, MA, Brasil; 2 Hospital Universitário da Universidade Federal do Maranhão, Universidade Federal do Maranhão, São Luís, MA, Brasil; 3 Programa de Pós Graduação em Saúde Coletiva, Universidade Federal do Maranhão, São Luís, MA, Brasil; 4 Departamento de Pediatria, Faculdade de Medicina da USP, Universidade de São Paulo, São Paulo, SP, Brasil; 5 Departamento de Puericultura e Pediatria, Faculdade de Medicina de Ribeirão Preto, Universidade de São Paulo, Ribeirão Preto, SP, Brasil; 6 Departamento de Clínica Infantil, Faculdade de Odontologia de Ribeirão Preto, Universidade de São Paulo, Ribeirão Preto, SP, Brasil; 7 Departamento de Saúde Pública, Universidade Federal do Maranhão, São Luís, MA, Brasil; 8 Departamento de Medicina I, Universidade Federal do Maranhão, São Luís, MA, Brasil

**Keywords:** Breast feeding, Weaning, Risk factors, Exclusive breastfeeding, Early supplementary feeding

## Abstract

The introduction of early complementary feeding (ECF) is determined by different factors depending on when it occurs. The objective of this study was to analyze factors associated with the introduction of ECF in two different moments of the infant's life: from zero to three and from four to five months of age. A cohort with 3,306 dyads studied in the BRISA survey in São Luis/MA in 2010 was used. Questionnaires were applied at birth and at follow-up when the infants were 15 to 36 months of age of women with more than 20 weeks of gestational age, residing in this municipality. A multivariate model of multinomial logistic regression was used to verify associations between independent variables and ECF at 0 to 3 months and at 4 to 5 months of age. A hierarchical analysis model was used to select variables for confounding adjustment. Variables with a P-value <0.05 were considered significant. For ECF introduced between 0-3 months, the variables “use of pacifier”, “maternal paid activity”, “smoking”, and “postpartum pregnancy” were identified as risk factors. The variables “use of pacifier” and “maternal paid activity” remained associated as a risk for ECF introduced from 4-5 months. The variable ‘mother without partner’ (RR=1.26 and P=0.04) represented a risk factor for ECF only for the 4-5 months period. Although each period presented specific risk factors, the use of pacifier and maternal professional activity were associated in the two periods studied, indicating their importance for the introduction of ECF.

## Introduction

Exclusive breastfeeding (EBF) is recommended by the World Health Organization until six months of age, and until two years of age, breastfeeding in addition to complementary feeding (CF) is advised (1). Studies show that human milk is superior to that of other species and can prevent infant death, diarrhea, allergies, respiratory infections, hypertension, hypercholesterolemia, and diabetes. Furthermore, it reduces the chance of obesity and might have a positive effect on intelligence and on the affective bond between mother and child (1,2).

In Brazil, the prevalence of EBF in children under 6 months in capital cities is considered low (41%) (3). In the Northeast region of the country, this prevalence is even lower (37%) (3).

The introduction of CF at early ages (ECF) is characterized by the association of other foods to breastfeeding before the first six months of life (1,3). It is considered a counterpoint to the benefits of EBF, since it is known that the baby has physiological and neurological maturity to receive other foods only after six months (1).

The Brazilian National Survey of Demography and Child and Women Health has shown that the introduction of non-human milk is high even in children under two months (18%) and about one-third of children are weaned between the fourth and fifth months (4).

The hypothesis of this study was that different factors determine when ECF is introduced in the infant's life since different situations are experienced by the mothers at three months and from four to six months of the infant’s life. The return to work after maternity leave can be cited as an example (5).

Although the risk factors associated with ECF are widely addressed in the literature (6), few studies attempt to clarify the conditions in which it occurs, considering different periods during the first six months (7–09). In addition, population-based studies can provide a better understanding of this context.

The present study used multinomial analysis in a cohort of children followed from birth in a capital of the Brazilian Northeastern region to investigate the determinants of the ECF introduction in two distinct periods: the first 3 months and the fourth and fifth months of life of the infant.

## Material and Methods

This cohort study is based on the research “Etiological factors of preterm birth and consequences of perinatal factors on child health: birth cohorts in two Brazilian cities - BRISA” developed in the cities of São Luis, capital of Maranhão state and Ribeirão Preto, São Paulo state. In this research, only data from the São Luis birth cohort were used. Data were obtained at the time of birth and at follow-up when children had from 15 to 36 months of life, making 3,215 dyads.

**Figure 1:**
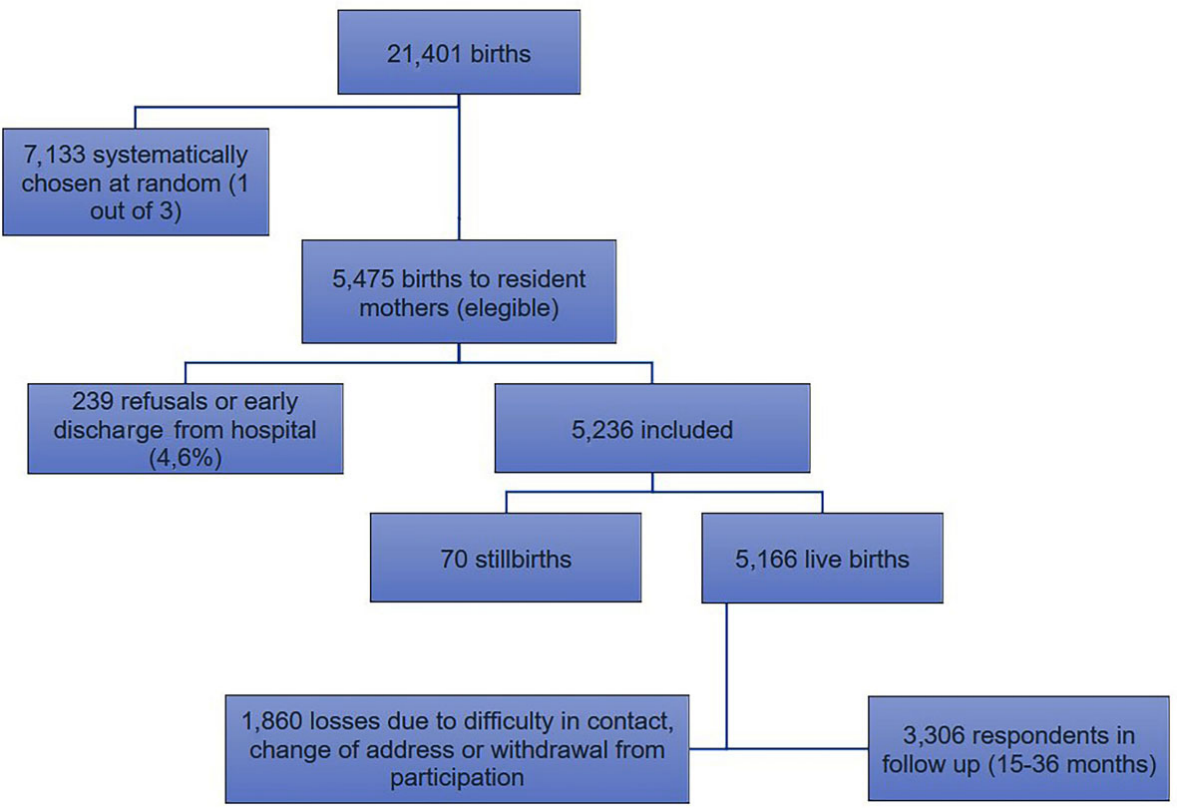
Sample flowchart of the BRISA birth cohort in São Luis, MA, Brazil, 2010–2013.

The BRISA birth cohort in São Luis sought to evaluate a third of the 21,401 births that occurred in the municipality in 2010. The sample was stratified by maternity centers excluding those with less than 100 births per year, in a sample proportional to the number of births. The minimum sample size was set at 5000. From the 7,133 women systematically randomized, 5,475 were considered eligible for the study and the sample consisted of 5,236 postpartum women and, after the exclusion of 70 stillbirths, 5,166 were effectively interviewed at birth. From this total, 3,306 (63.99% of the sample) were recovered at 15-36 months of age and the other 1,860 (36%) children were considered as losses for this follow-up, due to difficulty in contacting, change of address, or withdrawal from participation. The flowchart in Figure 1 illustrates the sample from the birth cohort. Silva et al. (10) published details on the BRISA cohort methodology.

The data referring to the mother and/or the person responsible for the child were collected from January to December 2011. Those referring to the newborns were obtained at the second and third years of life, from April 2011 to March 2013.

The outcome variable (ECF) was obtained from the question “Until what age was your child exclusively breastfed?” (what EBF consisted of was explained to the mother). The answer had three categories: 0-3 months, 4-5 months, and 6 or more months of life, which was used as reference. The explanatory variables referred to socioeconomic and demographic characteristics, and maternal life habits; socioeconomic, demographic, perinatal, and routine characteristics of the child; and characteristics of EBF and eating habits of newborns.

Data analysis was performed using Stata^®^ 10.0 statistics package (StataCorp, USA). A descriptive analysis of the characteristics of ECF was performed. The normality of the data distribution was tested by Shapiro Wilk test.

Multinomial logistic regression analysis was used in order to evaluate associations between the period of ECF introduction and the independent variables. As this was a longitudinal study, we used relative risk, which is the appropriate measure.

**Figure 2:**
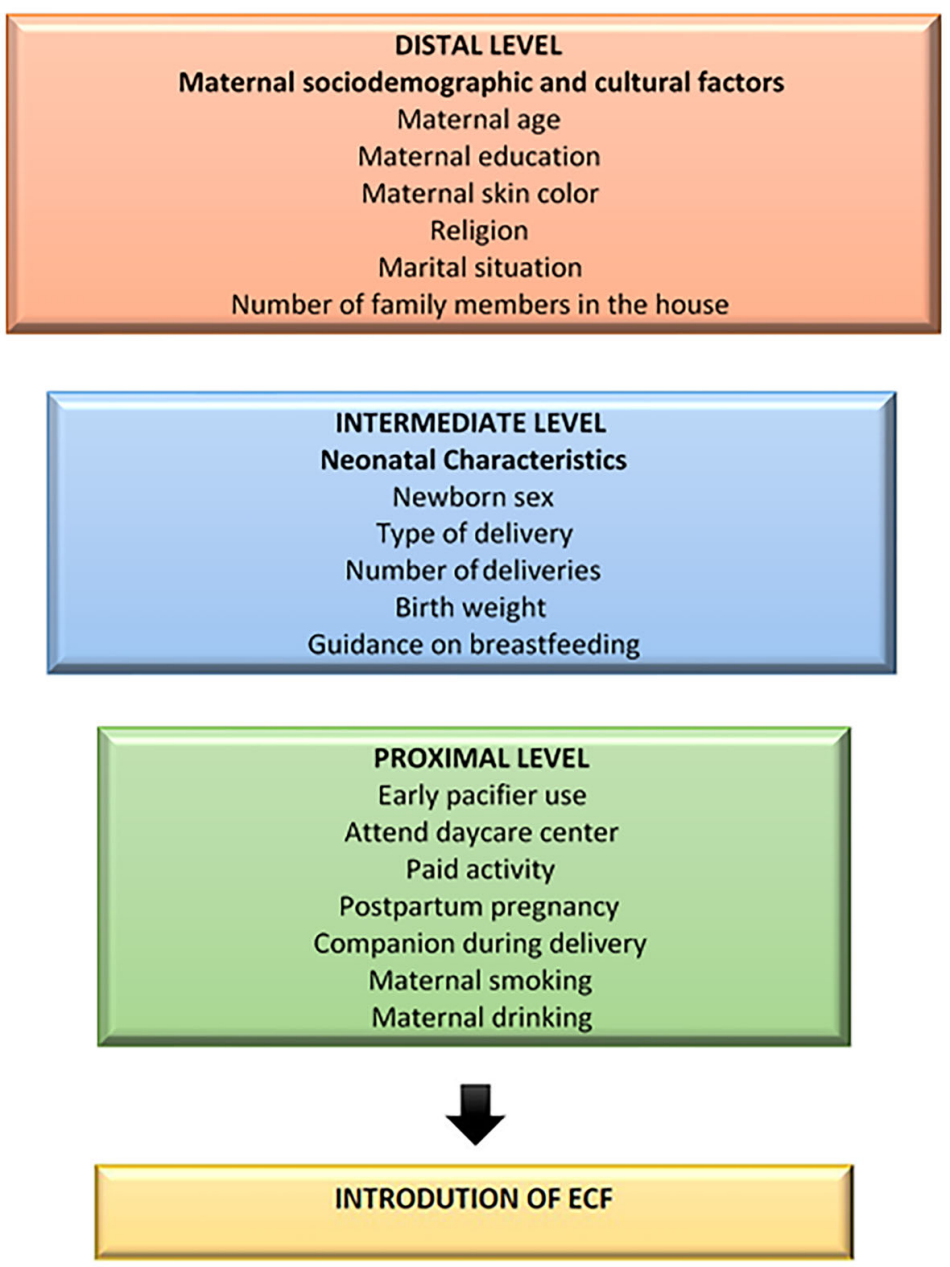
Hierarchical theoretical model. ECF: early complementary feeding.

The choice of variables to compose the multinomial regression model was established by a hierarchical analysis in which the introduction of ECF (yes/no) (at any point in the first six months) was used as the outcome variable. For this analysis, the independent variables were categorized into three blocks organized according to influence on the outcome. The variables age, schooling, self-reported maternal skin color, marital status, religion, and number of relatives in cohabitation composed the distal block. The intermediate block was constituted by the variables gender, birth weight, type of delivery, number of births, presence of companion at delivery, and orientation on breastfeeding. The variables postpartum pregnancy, paid activity, smoking, alcoholism, day care, and early use of pacifier were in the proximal block (Figure 2).

The criterion for variable inclusion in each block was established through a conceptual framework based on evidence from the literature. Since the first block variables presented P<0.05, they were maintained in the following blocks.

The BRISA survey was approved by the University Hospital of the Federal University of Maranhão Committee on Ethics in Research (opinion No. 223/2009), in accordance with Resolution CNS 196/96, in force at the time.

## Results

At birth, mothers’ mean age was 25.4±6.02 years, 8.6% had a partner, 62.6% studied until high school, and 67.9% had black/brown skin color. Type of delivery was 51.2% vaginal, and 47.9% of the women were primiparous. The other socioeconomic characteristics and maternal life habits are described in Table 1.

**Table 1:** Socioeconomic, demographic, and maternal life habits characteristics in São Luis, MA, Brazil, 2010-2013.

Variables	n	%
Maternal age (years)		
<19	589	17.8
19–35	2436	73.7
>35	281	8.5
Maternal education		
Elementary School	771	23.4
High school	2066	62.6
Higher education	462	14.0
Self-reported maternal skin color		
White	578	17.5
Black	437	13.2
Brown	2245	67.9
Other	42	1.4
Religion		
Yes	2742	82,9
No	564	17.1
Marital status		
With a partner	2665	80.6
Without a partner	641	19.4
Paid activity		
Yes	1131	34.2
No	2175	65.8
Smoking		
Yes	162	4.9
Not	3112	95.1
Alcoholism		
Yes	36	1.1
No	3269	98.9
Type of delivery		
Normal	1691	51.2
Cesarean section	1615	48.8
No. deliveries		
1	1585	47.9
2–4	1624	49.2
>4	97	2.9
Accompanying in childbirth		
Yes	932	28.5
No	2342	71.5
No. of relatives in cohabitation		
0 (zero)	1883	57.0
1–3	926	28.0
4+	495	15.0
Guidance on breastfeeding		
Yes	2249	68.9
No	1013	31.1
Postpartum pregnancy		
Yes	291	8.9
No	2984	91.1

Fifty percent of the children were male and 92.3% were born with 2,500 g or more. The vast majority (96.4%) did not attend day care center and 20.1% started using pacifiers before the age of 3 months (data not shown).

**Figure 3:**
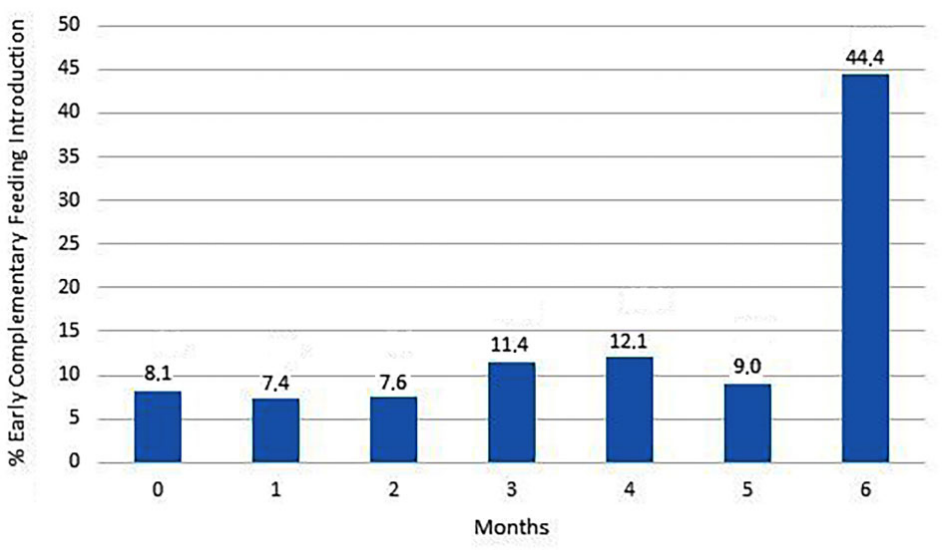
Distribution of the introduction of early complementary feeding in the first six months of life in São Luis, MA, 2010–2013.

EBF until 5 months of life was done in 55.6% of the children, which means that ECF occurred in 44.4% of the cases. The distribution of ECF introduction in the first 6 months is shown in Figure 3. It is worth noting the introduction of semisolid or solid foods in the infants’ feeding in early periods, although in small percentages (Table 2).

**Table 2:** Breastfeeding and feeding habits of babies from São Luis, MA cohort, Brazil, 2010-2013.

Variables	Mean±SD	n	%
Introduction of ECF	4.59±2.27		
0–3 months		1104	34.5
4–5 months		677	21.1
6–7 months		1423	44.4
Total		3204	100.0
Introduction of liquids	5.43±1.56		
0–3 months		277	13.9
4–5 months		406	20.4
6–7 months		1308	65.7
Total		1991	100.0
Formula introduction	4.24±2.75		
0–3 months		483	32.5
4–5 months		418	28.1
6–7 months		585	39.4
Total		1486	100.0
Introduction of solid and semi-solid foods	5.85±1.00		
0–3 months		112	4.1
4–5 months		401	14.6
6–7 months		2235	81.3
Total		2748	100.0

ECF: early complementary feeding; SD: standard deviation.

Table 3 presents the hierarchical analysis of the variables of the three structured blocks in relation to the outcome variable (ECF - yes/no). Only the variables associated with ECF with statistical significance were maintained in Table 4.

**Table 3:** Hierarchical theoretical model of statistically significant variables associated to early complementary feeding in São Luis, MA, Brazil, 2010-2013.

	Early complementary feeding											
	Distal Block				Intermediate Block				Proximal Block			
	OR	95%CI		P	OR	95%CI		P	OR	95%CI		P
Self-reported maternal skin color												
White	1.48	1.15	1.92	**0.003**	1.32	1.09	1.60	**0.004**	1.24	1.02	1.50	**0.007**
Black	COL				-	-	-	-	-	-	-	-
Brown	1.18	0.96	1.46	0.119	-	-	-	-	-	-	-	-
Other	1.41	0.71	2.78	0.325	-	-	-	-	-	-	-	-
Marital status												
With a partner	Ref	-	-	-	-	-	-	-	-	-	-	-
Without a partner	1.22	1.02	1.45	**0.028**	1.22	1.03	1.45	**0.024**	1.23	1.03	1.46	**0.020**
No. of deliveries												
1					Ref	-	-	-	-	-	-	-
2-4					-	-	-	-	-	-	-	-
>4					0.62	0.40	0.96	**0.034**	0.69	0.45	1.06	0.093
Paid Activity												
Yes					-	-	-	-	1.37	1.17	1.59	**0.000**
No					Ref	-	-	-	-	-	-	-
Postpartum pregnancy												
Yes									1.30	1.01	1.69	**0.045**
No									Ref	-	-	-
Smoking												
Yes									2.26	1.55	3.29	**0.000**
No									Ref	-	-	-
Early use of pacifiers												
Yes									2.75	2.25	3.35	**0.000**
No									Ref	-	-	-

OR: odds ratio; Ref: reference category; 95%CI: confidence interval; COL: collinear. Bold type indicates statistical significance.

**Table 4:** Multinomial analysis for risk factors for introduction of early complementary feeding (ECF) of infants at 0-3 and 4-5 months of age in São Luis, MA, Brazil, 2010-2013.

Determinants	ECF at 0 to 3 months			ECF at 4-5 months		
	Adjusted analysis*			Adjusted analysis*		
	RR	95%CI	P	RR	95%CI	P
Marital status						
With a partner	Ref	-	-	-	-	-
Without a partner	1.21	0.99-1.47	0.06	1.26	1.01-1.57	**0.04**
Paid activity						
Yes	Ref	-	-	-	-	-
No	1.37	1.16-1.63	**<0.01**	1.42	1.17-1.72	**<0.01**
Postpartum pregnancy						
Yes	1.37	1.02-1.83	**0.03**	1.25	0.90-1.75	0.18
No	Ref	-	-	-	-	-
Smoking						
Yes	2.81	1.90-4.17	**<0.01**	1.44	0.85-2.36	0.15
No	Ref	-	-	-	-	-
Early use of pacifier						
Yes	3.46	2.80-4.28	**<0.01**	1.77	1.37-2.29	**<0.01**
No	Ref	-	-	-	-	-

RR: relative risk; Ref: reference category; *adjusted for self-reported maternal skin color, marital status, number of deliveries, paid activity, postpartum pregnancy, smoking, and early use of pacifier. Bold type indicates statistical significance.

In the multinomial analysis, the variables ‘paid activity', ‘smoking', ‘early pacifier use', and ‘postpartum pregnancy’ were risk factors for ECF start within the first 3 months of life (Table 4). For the 4 to 5-month period, the variables ‘early pacifier use’ and ‘having paid activity’ remained associated as risk factors for ECF introduction. The variable ‘mother without partner’ represented a risk factor for ECF only in this period (Table 4).

## Discussion

In the present study, we demonstrated that the main determinants of weaning at 0-3 months were a new pregnancy and the presence of maternal smoking. Specifically associated to 4th and 5th months is the absence of a partner. Finally, the factors “early use of pacifiers” and “mothers with paid activity” were associated to both the first and the second periods analyzed.

In São Luis, the mean duration of EBF was 4.6 months, showing that the introduction of CF occurred earlier than what is recommended by WHO. This was similar to the average of 4 months for CF found in Belo Horizonte (11), but contrasts with data obtained in Bahia, another state of Northeastern Brazil (mean of 2.5 months) (12). These results show great variability, probably due to the influence of different regional socioeconomic and cultural aspects related to the supply of other foods (13).

The early use of a pacifier constituted a risk factor for the introduction of CF in both early (up to 3 months) and late (4 to 5 months) periods. These findings corroborate several studies that demonstrated a significant association between early use of the pacifier and ECF (14–16). Over the first three months, several factors might influence the provision of a pacifier to calm the baby, such as the mother's difficulties with breastfeeding, the perception of poor milk production (15), and the anxiety and insecurity about the child's feeding process, which can consequently lead to the introduction of ECF (17).

It is believed that the use of pacifiers decreases nipple suckling causing less stimulation of the nipple-areolar complex, which implies less milk production, generating the need for feeding complementation (18). However, there is no consensus in the literature regarding the effects of pacifiers on breastfeeding (19). Studies speculate that such a habit would not be an isolated cause for the introduction of CF, which would instead be explained by cultural factors, or factors linked to the institutional care of the puerperal woman and to the mother's conception in relation to breastfeeding (20).

From the fourth month onwards, the introduction of CF can be influenced by factors such as maternal return to work and the need for the child to stay with other caregivers or in institutions; in such cases, a pacifier may be used as a reinforcement to weaning (21). Maternal paid activity was a risk for the introduction of CF within 3 months and in the 4th and 5th months. In the US, evidence shows that when the mother returns to work there is a marked decline in the intention to exclusively breastfeed her baby (22). In a cohort conducted in São Paulo, mothers who did not work outside the home had a later introduction of CF (23).

Other elements may influence the relationship between paid work and the introduction of ECF. The lack of support from institutions, unfavorable conditions for breastfeeding, and distance between mother workplace and child were the main difficulties pointed out by mothers regarding the maintenance of breastfeeding (23). Also, when the mother has to deal with domestic tasks other than her external occupation, ECF has a greater chance of being established (3,20).

However, favorable conditions for the maintenance of breastfeeding in maternal work may favor EBF until the sixth month. In Chile, Valdés et al. (24) reported a successful experience of EBF maintenance until the sixth month among mothers who returned to work. The intervention included early counseling and monthly clinical monitoring of the mother and baby.

In this study, maternal smoking during lactation was associated with the introduction of CF earlier in life. There is no consensus in the literature on the risks of weaning and maternal smoking. Some studies did not find this association (25) while others pointed to smoking as a cause for the introduction of CF (26), probably due to the reduction of basal prolactin with a decrease in milk production and a change in milk composition and taste (27–29).

An association between the occurrence of a new pregnancy and the introduction of CF was also found. We did not find epidemiological studies exploring this association in the literature. However, studies with a qualitative approach have pointed out possible causes for this phenomenon based on women's cultural aspects (30). Issues such as the fear of going into early labor or having a miscarriage, the idea that breastfeeding will steal the nutrients of the fetus, mother's perception of fatigue, besides the discomfort caused by the hormones of pregnancy (31) may facilitate ECF, especially in the first months of pregnancy.

This study pointed to a greater risk for ECF between the 4th and 5th month among women who did not have a partner compared to married women. This finding corroborates the studies of Morgado et al. (32) and Resende et al. (33) that emphasize the fathers’ involvement in the duration of breastfeeding. These studies suggest that this support network acts as reinforcement for the prolongation of EBF, thus postponing ECF (32–34). Bernardi et al. (34) also demonstrated that unmarried women were more likely to introduce ECF.

The father's understanding that breastfeeding would favor the child's health may determine collaborative behaviors, such as taking on household chores. These attitudes can contribute positively to avoiding or delaying the introduction of ECF (25). However, day-to-day difficulties in caring for the baby can be tiring, causing the partner to eventually give up this routine.

The use of a recall questionnaire represented a limitation in this study as it may have compromised the accuracy of some information. The absence of important variables for this subject, such as breastfeeding in the first hour of life, was also a limitation. Nevertheless, the cohort design, the populational character, and the large sample size allowed great inference power. In addition, using multinomial regression analysis made it possible to study associations for introduction of ECF in two different periods of a baby's life that possibly represent different realities and motivations.

Two variables were associated with weaning in the two periods studied: the early use of pacifiers and maternal paid work outside the home. This suggested, on the one hand, the need to change a habit of social and emotional origin that can only be modified through health education actions. On the other hand, it points out the need to provide guidance to breastfeeding mothers on how to maintain EBF and guarantee physical and organizational conditions at work that favor the maintenance of breastfeeding. The introduction of ECF in the first 3 months of life was associated with a new pregnancy and smoking during pregnancy. Only the absence of a partner was specifically associated with weaning after the child's 4th month of life, when parental and family support can make a difference in avoiding the introduction of ECF.
